# Associated factors of primary snoring and obstructive sleep apnoea in patients with sleep bruxism: A questionnaire study

**DOI:** 10.1111/joor.13354

**Published:** 2022-07-12

**Authors:** Zhengfei Huang, Ghizlane Aarab, Thiprawee Chattrattrai, Naichuan Su, Catherine M. C. Volgenant, Antonius A. J. Hilgevoord, Nico de Vries, Frank Lobbezoo

**Affiliations:** ^1^ Department of Orofacial Pain and Dysfunction, Academic Center for Dentistry Amsterdam (ACTA) University of Amsterdam and Vrije Universiteit Amsterdam Amsterdam The Netherlands; ^2^ Department of Clinical Neurophysiology OLVG Amsterdam The Netherlands; ^3^ Department of Oral Public Health, Academic Center for Dentistry Amsterdam (ACTA) University of Amsterdam and Vrije Universiteit Amsterdam Amsterdam The Netherlands; ^4^ Department of Preventive Dentistry, Academic Center for Dentistry Amsterdam (ACTA) University of Amsterdam and Vrije Universiteit Amsterdam Amsterdam The Netherlands; ^5^ Department of Otorhinolaryngology ‐ Head and Neck Surgery OLVG Amsterdam The Netherlands; ^6^ Department of Otorhinolaryngology ‐ Head and Neck Surgery Antwerp University Hospital (UZA) Antwerp Belgium

**Keywords:** associated factor, clinical study, obstructive sleep apnoea, primary snoring, sleep bruxism

## Abstract

**Background:**

By being aware of the associated factors of primary snoring (PS) and obstructive sleep apnoea (OSA) in sleep bruxism (SB) patients, dentists may contribute to the screening and early recognition of SB patients with PS or OSA.

**Objective:**

To identify the associated factors of PS and OSA from questionnaire‐based data in SB patients.

**Methods:**

A total of 968 self‐reported SB patients (31.6% men; median age 44.5 years) were retrospectively enrolled. Self‐reported sleep‐related breathing status (viz., no sleep‐related breathing condition, PS and OSA) was the dependent variable. Independent variables were questionnaire‐based data on demographics, lifestyle, psychological status, pain and sleep.

**Results:**

For PS, no statistically significant associated factor was identified in analyses. For OSA, increased age (OR = 1.04 [1.03–1.06]), male gender (OR = 3.33 [2.17–5.00]), daily alcohol consumption (OR = 1.96 [1.18–3.33]), depression (OR = 1.10 [1.06–1.14]), daytime sleepiness (OR = 2.94 [1.85–4.76]) and high risk of gastroesophageal reflux disease (GERD; OR = 2.63 [1.52–4.76]) were found to be significant risk factors, while high risk of temporomandibular disorder (TMD) pain (OR = 0.51 [0.30–0.86]) and chronic pain (OR = 0.73 [0.59–0.90]) were significant protective factors. These results were confirmed in the subsequent network analysis.

**Conclusion:**

Within the limitations of this study, no associated factor is identified for PS. For OSA, dentists should keep in mind that increased age, male gender, daily alcohol consumption, depression, daytime sleepiness and high GERD risk are associated with increased OSA risk in SB patients, while high TMD‐pain risk and chronic pain are associated with decreased OSA risk in this population.

## 1 INTRODUCTION

Obstructive sleep apnoea (OSA) is a common sleep disorder with an estimated prevalence of 22% in men and 17% in women.^1^ OSA is characterised by repetitive partial (hypopnoea) or complete (apnoea) collapse of the upper airway during sleep, which may consequently lead to oxygen desaturation, respiratory arousals and non‐restorative sleep.^1^ In addition, OSA has also been reported to be associated with systemic diseases, such as diabetes.^2^ Snoring is a common sleep‐related breathing condition that is characterised by audible vibrations of the upper airway during respiration.^3^ As a social nuisance that may lead to impaired sleep quality of bedpartner and marital disharmony,^4^ snoring is one of the most frequently reported symptoms of OSA. Snoring can also occur without OSA and is then called primary snoring (PS). An apnoea‐hypopnoea index (AHI) of five events per hour is the threshold to distinguish between PS (AHI <5) and OSA (AHI≥5).^5^

As a sleep condition that has been reported to be associated with both OSA and PS,^6^, ^7^ sleep bruxism (SB) was found to affect approximately 13% of the general adult population.^8^ SB is defined as ‘masticatory muscle activity during sleep that is characterized as rhythmic (phasic) or non‐rhythmic (tonic) and is not a movement disorder or a sleep disorder in otherwise healthy individuals’.^9^ Despite the fact that polysomnography (PSG) is the gold standard to diagnose SB, dentists recognise patients with SB mainly based on patients' self‐report (viz., self‐awareness of clenching/grinding during sleep and self‐reported discomfort of the jaw and/or jaw muscle) and clinical examination (e.g., the presence of excessive tooth wear and/or masseter hypertrophy).^9^ The reported prevalence of OSA in patients with SB is high and ranges from 20.6%–27.3%.^7^, ^10^, ^11^ The prevalence of PS in SB patients, on the other hand, is still unclear, but a previous study found that SB patients without OSA experienced more snoring events during sleep than healthy individuals.^6^ In addition, snoring as a symptom was reported in 32.7% of SB patients and was identified as a risk factor of SB in the general population.^12^ Taken together, these findings suggest that patients with SB are at high risk of PS and OSA.

Factors like age, gender, body mass index (BMI), depression and temporomandibular disorder (TMD) have been reported to be associated with PS and/or OSA in previous studies.^13^, ^14^, ^15^ However, to the best of the authors' knowledge, no published study has investigated the associated factors of PS and OSA in SB patients. By being aware of the associated factors of PS and OSA in SB patients, dentists may contribute to the screening and early recognition of patients with PS or OSA, especially in countries where individuals visit their dentists on a regular basis. By referring patients with suspected PS and/or OSA to sleep physicians, dentists may further facilitate the early management of PS and OSA, thereby preventing the potential psychological and physical consequences of these sleep‐related breathing conditions. Therefore, the aim of this study was to identify associated factors of PS and OSA from among an extensive collection of self‐reported questionnaire data on demographics, lifestyle, psychological status, pain and sleep in SB patients. We hypothesized that, in addition to the frequently reported associated factors of PS and OSA (e.g., age and gender), several other factors/conditions that have been reported to be associated with SB, PS and OSA, such as mental health conditions (e.g., depression and anxiety)^14^, ^16^ and TMD,^15^, ^17^ may also be identified as associated factors in this specific population.

## 2 METHODS

### 2.1 Participant recruitment

Participants were selected from among patients who were referred to the clinic for Orofacial Pain and Dysfunction (OPD) of the Academic Centre for Dentistry Amsterdam (ACTA), Amsterdam, The Netherlands, between January 2017 and December 2020. The reasons of referring patients to the OPD clinic include tooth wear, OSA/snoring and TMD/oro‐facial pain. Prior to the first appointment at the OPD clinic, all referred patients were asked to fill in a set of questionnaires that were used as part of the regular intake procedure. Among others, these questionnaires included questions about patients' demographics, life style, psychological status, pain‐related complaints and sleep‐related complaints. In addition to the regular set of questionnaires, as part of the standard care procedure of the OPD clinic, dedicated questionnaires were completed by patients with specific complaints. For example, for patients who reported SB, the Oral Behaviours Checklist (OBC), a self‐report scale to quantify the frequency of jaw behaviours,^18^ was asked to complete. Adult patients who consented to the use of their questionnaire data for scientific purposes and answered ‘1–3 nights/week’ or ‘4–7 nights/week’ to the first question of the OBC (viz., ‘Clench or grind teeth when asleep, based on any information you may have’) were identified as patients with SB and were included in this retrospective study. Patients who were younger than 18 years old were excluded. The protocol of this retrospective study was approved by the Medical Ethics Committee of ACTA (file number 201986) and the committee stated that the Dutch law on Research Involving Human Subjects Act (WMO) did not apply to this study.

### 2.2 Dependent variable

Sleep‐related breathing status (viz., no sleep‐related breathing condition, PS and OSA) was the dependent variable in this study. As such, participants were categorised as participants without sleep‐related breathing condition, participants with probable primary snoring (primary snorers), or participants with probable OSA (OSA patients). The STOP‐Bang (snoring, tiredness, observed apnoea, high blood pressure, body mass index, age, neck circumference, gender) questionnaire^19^ is a validated and widely accepted screening tool for OSA. In the original study on the STOP‐Bang questionnaire, the authors suggested to use answering ‘Yes’ to two questions as the cut‐off to distinguish between ‘Low OSA risk’ (viz., answering ‘Yes’ to 0–2 questions) and ‘High OSA risk’ (answering ‘Yes’ to 3–8 questions) due to the best combination of sensitivity and specificity of this criterion.^19^ At the OPD clinic, only patients who reported snoring and other OSA‐related symptoms were asked to complete the STOP‐Bang questionnaire. Hence, participants who were not asked to complete the STOP‐Bang questionnaire were identified as participants without sleep‐related breathing condition. Participants who answered ‘No’ to the first question of STOP‐Bang (‘Do you snore loudly’?) and were identified as ‘Low OSA risk’ based on the interpretation criteria of STOP‐Bang were also categorised as participants without sleep‐related breathing condition. Participants who answered ‘Yes’ to the snoring question but were identified as ‘Low OSA risk’ were categorised as primary snorers. Participants who were identified as ‘High OSA risk’ were categorised as OSA patients.

### 2.3 Independent variables

In this study, independent variables were divided into five categories:

1. Demographic variables: age and gender;
2. Lifestyle variables: smoking, daily alcohol consumption and intake of sleep medication;
3. Psychological variables: somatic symptoms, depression, anxiety and stress;
4. Pain‐related variables: temporomandibular disorder (TMD) pain, TMD‐related headache (temple area), primary headache (migraine or tension type) and chronic pain (intensity and disability);
5. Sleep‐related variables: daytime sleepiness, gastroesophageal reflux disease (GERD) and dry mouth.

All the above‐mentioned information was obtained from the set of questionnaires of the OPD clinic. Demographic variables and life style variables were obtained from a custom‐made questionnaire focusing on the patients' personal information. Data about somatic symptoms, depression and anxiety were obtained from the Patient Health Questionnaire (PHQ)‐15,^20^ the Patient Health Questionnaire (PHQ)‐9,^20^ and the Generalised Anxiety Disorder (GAD)‐7 questionnaire,^20^ respectively. Patients were also asked how much stress they experienced in the past month, as to assess daily stress. Among the pain‐related variables, data about TMD pain, primary headache (migraine or tension type) and chronic pain (intensity and disability) were obtained from the TMD‐pain screener,^20^ the Headache Screening Questionnaire (HSQ),^21^ and the Graded Chronic Pain Scale (GCPS),^20^ respectively. In addition, TMD‐related headache was evaluated using the 5th question (‘In the last 30 days, have you had any headaches that included the temple areas of your head’?) of the Symptom Questionnaire of the diagnostic criteria for temporomandibular disorders (DC/TMD).^20^ Sleep‐related variables, viz., daytime sleepiness, GERD and dry mouth, were obtained from the Epworth Sleepiness Scale (ESS),^22^ the Gastroesophageal reflux disease Questionnaire (GerdQ),^23^ and the Xerostomia Inventory (XI),^24^ respectively. Among the above‐mentioned questionnaires, PHQ‐15, PHQ‐9, GAD‐7, TMD screener and GCPS are embedded in the comprehensive DC/TMD Axis II instrument.^20^ The scoring criteria and interpretation of all independent variables are listed in Table 1.

**TABLE 1:** The response options and quantification of the independent variables

Demographic variables	
Age	As quantitative data (y)
Gender	0 = Female; 1 = Male
Life style variables	
Smoking	0 = No; 1 = Yes
Daily alcohol consumption	0 = No; 1 = Yes
Intake of sleep medication	0 = No; 1 = Yes
Psychological variables	
Somatic symptom (PHQ‐15)	As quantitative data (0–30)
Depression (PHQ‐9)	As quantitative data (0–27)
Anxiety (GAD‐7)	As quantitative data (0–21)
Stress	As quantitative data (0–4)
Pain‐related variables	
TMD pain (TMD‐pain screener)	0–3 = Low risk of TMD pain; 4–7 = High risk of TMD pain
TMD‐related headache (HSQ)	0 = No; 1 = Yes
Primary headache (migraine or tension type; DC/TMD)	0 = Neither migraine nor tension type headache; 1 = Probably migraine or tension type headache
Chronic pain (GCPS)	As quantitative data (0–4)
Sleep‐related variables	
Daytime sleepiness (ESS)	0–10 = No; 10–24 = Yes
GERD (GerdQ)	0–8 = Low risk of GERD; 9–18 = High risk of GERD
Dry mouth (XI)	As quantitative data (0–44)

Abbreviations: ESS, Epworth sleepiness scale; GAD, generalised anxiety disorder; GCPS, graded chronic pain scale; GERD, gastroesophageal reflux disease; GerdQ, gastroesophageal reflux disease questionnaire; HSQ, headache screening questionnaire; PHQ, patients health questionnaire; TMD, temporomandibular disorder; XI, xerostomia inventory.

### 2.4 Statistics

As descriptive statistics, nominal and ordinal data were presented as percentages. For continuous data, Kolmogorov–Smirnov test was first used to check if the data was normally distributed. Normally distributed data were presented as mean ± SD; data that were not normally distributed were presented as median (interquartile range; IQR). Multinomial logistic regression modelling was used to evaluate the association between the dependent variable (viz., sleep‐related breathing status: no sleep‐related breathing condition, PS and OSA) and the independent variables. As the first step, univariable logistic regression models were built to assess the association between the dependent variable and each of the independent variables. Independent variables that showed at least a weak association (*p* < .1) with the dependent variable were considered eligible to be entered in multivariable models. Before entering significant variables in the multivariable logistic regression model, multicollinearity was checked using a tolerance value <0.1 or variance inflation factor (VIF) >10.^25^ For the multivariable model, the significance level was set at *p* < .05. Analyses were conducted using the IBM SPSS Statistics 27 software package (IBM Corp.).

In addition, network analysis was performed in RStudio (version 1.2.1335, RStudio PBC), a programming software, using the package ‘bootnet’ (version 1.4.3)^26^ as to evaluate how all variables related to each other in a multivariable system. Unlike the multinomial logistic regression model, it is not necessary to specify the dependent variable and the independent variables, because the network model estimates the association between any two variables and controls for all the other variables in the network (i.e., the conditional dependence relationships). In this analysis, hyperparameter 𝛾 (gamma; the key parameter in network analysis) was set to 0.5 to reduce the chance of including false positive associations in the network. By using network analysis, indirect associations between factors can be estimated and bridge factors (i.e., factors that connect two other factors) can also be identified. The visualisation of the network was achieved using the package ‘qgraph’ (version 1.6.5).^27^ Finally, the robustness of the network was evaluated using the non‐parametric bootstrapping method, which repeatedly estimates the model based on simulated data.^26^

## 3 RESULTS

Of the 2218 patients who completed the OBC from January 2017 to December 2020, 1024 patients reported to grind their teeth more than one night per week. Of these 1024 patients, 56 adolescents were excluded so that finally 968 adult patients were included in this study. Of the 968 participants, 726 (75%) were categorised as participants without sleep‐related breathing condition, 71 (7.3%) were categorised as primary snorers, and 171 (17.7%) were categorised as OSA patients. Descriptive statistics of the participants are shown in Table 2.

**TABLE 2:** Descriptive statistics of independent variables in participants without sleep‐related breathing condition, primary snorers and OSA patients

	Total (*N* = 968)	Participants without sleep‐related breathing condition (N = 726)	Primary snorers (*N* = 71)	OSA patients (*N* = 171)
Demographic variables				
Age (y, median [IQR])	44.5 (33–54)	43 (31–53)	45 (37–51)	51 (43–57)
Male gender (*N*, %)	306 (31.6%)	187 (25.8%)	15 (21.1%)	104 (60.8%)
Lifestyle variables				
Smoking (*N*, %)	161 (16.6%)	122 (16.8%)	11 (15.5%)	28 (16.4%)
Daily alcohol consumption (*N*, %)	108 (11.2%)	67 (9.2%)	3 (4.2%)	38 (22.2%)
Intake of sleep medication (*N*, %)	67 (6.9%)	51 (7.0%)	4 (5.6%)	12 (7.0%)
Psychological variables				
Somatic symptoms (median [IQR])	8 (4–11)	7 (4–11)	9 (4–14)	8 (5–11)
Depression (median [IQR])	5 (2–8)	4 (2–8)	5 (3–8)	7 (3–10)
Anxiety (median [IQR])	3 (1–7)	3 (1–7)	3 (1–6)	4 (1–7)
Stress (median [IQR])	1 (1–2)	1 (1–2)	1 (1–2)	1 (1–2)
Pain‐related variables				
High risk of TMD pain (*N*, %)	564 (58.3%)	468 (64.5%)	45 (63.4%)	51 (29.8%)
TMD‐related headache (*N*, %)	385 (39.8%)	311 (42.8%)	32 (45.1%)	42 (24.6%)
Probably migraine or tension type (*N*, %)	635 (65.6%)	493 (67.9%)	48 (67.6%)	94 (55.0%)
Chronic pain (median [IQR])	1 (0–2)	1 (0–3)	1 (0–3)	0 (0–1)
Sleep‐related variables				
Daytime sleepiness (*N*, %)	166 (17.1%)	99 (13.6%)	10 (14.1%)	57 (33.3%)
High risk of GERD (*N*, %)	92 (9.5%)	47 (6.5%)	8 (11.3%)	37 (21.6%)
Oral dryness (median [IQR])	0 (0–9)	0 (0–10)	0 (0–13)	0 (0–3)

Abbreviations: GERD, gastroesophageal reflux disease; IQR, interquartile range; OSA, obstructive sleep apnoea; TMD, temporomandibular disorder.

The results of the logistic regression analyses are shown in Table 3. For PS, no statistically significant associated factor was identified when no sleep‐related breathing condition was the reference category. For OSA, age (OR = 1.04 [1.03–1.06]), male gender (OR = 3.33 [2.17–5.00]), daily alcohol consumption (OR = 1.96 [1.18–3.33]), depression (OR = 1.10 [1.06–1.14]), daytime sleepiness (OR = 2.94 [1.85–4.76]) and high risk of GERD (OR = 2.63 [1.52–4.76]) were found to be risk factors, while high risk of TMD pain (OR = 0.51 [0.30–0.86]) and chronic pain (OR = 0.73 [0.59–0.90]) were found to be protective factors.

**TABLE 3:** Univariable logistic regression model and multinomial logistic regression model of independent variables associated with PS and OSA

	Univariable logistic regression model				Multivariable logistic regression model			
	PS vs no sleep‐related breathing condition		OSA vs no sleep‐related breathing condition		PS vs no sleep‐related breathing condition		OSA vs no sleep‐related breathing condition	
Independent variables	*p* Value	OR (95% CI)	*p* Value	OR (95% CI)	*p* Value	OR (95% CI)	*p* Value	OR (95% CI)
Demographic variables								
Age	0.25	1.01 (0.99–1.03)	<.01	1.04 (1.03–1.06)			<.01	1.04 (1.03–1.06)
Gender								
Female	Reference							
Male	0.39	0.77 (0.43–1.39)	<.01	4.55 (3.13–6.25)			<.01	3.33 (2.17–5.00)
Lifestyle variables								
Smoking								
No	Reference							
Yes	0.78	0.91 (0.46–1.79)	.89	0.97 (0.62–1.52)				
Daily alcohol consumption								
No	Reference							
Yes	0.17	0.43 (0.13–1.41)	<.01	2.78 (1.82–4.35)			.01	1.96 (1.18–3.33)
Intake of sleep medication								
No	Reference							
Yes	0.66	2.62 (0.28–2.27)	1.00	1.00 (0.52–1.92)				
Psychological variables								
Somatic symptoms	0.15	1.04 (0.99–1.08)	.94	1.00 (0.97–1.03)				
Depression	0.22	1.03 (0.98–1.08)	<.01	1.06 (1.03–1.10)			<.01	1.10 (1.06–1.14)
Anxiety	0.84	0.99 (0.94–1.05)	.42	1.02 (0.98–1.05)				
Stress	0.81	0.97 (0.78–1.22)	.31	0.92 (0.79–1.08)				
Pain‐related variables								
TMD pain								
Low risk	Reference							
High risk	0.86	0.95 (0.57–1.59)	<.01	0.23 (0.16–0.34)			.01	0.51 (0.30–0.86)
TMD‐related headache								
No	Reference							
Yes	0.72	1.10 (0.67–1.79)	<.01	0.43 (0.30–0.63)			.60	0.86 (0.50–1.47)
Headache (migraine or tension type)								
No	Reference							
Yes	0.96	0.99 (0.58–1.67)	<.01	0.58 (0.41–0.81)			.58	1.14 (0.72–1.82)
Chronic pain	0.49	0.94 (0.79–1.12)	<.01	0.57 (0.48–0.66)			<.01	0.73 (0.59–0.90)
Sleep‐related variables								
Daytime sleepiness								
No	Reference							
Yes	0.92	1.04 (0.52–2.08)	<.01	3.13 (2.17–4.55)			<.01	2.94 (1.85–4.76)
GERD								
Low risk	Reference							
High risk	0.13	1.82 (0.83–4.00)	<.01	4.00 (2.50–6.25)			<.01	2.63 (1.52–4.76)
Oral dryness	0.21	1.02 (0.99–1.05)	.17	0.99 (0.96–1.01)				

Abbreviations: GERD, gastroesophageal reflux disease; OSA, obstructive sleep apnoea; PS, primary snoring; TMD, temporomandibular disorder.

**FIGURE 1:**
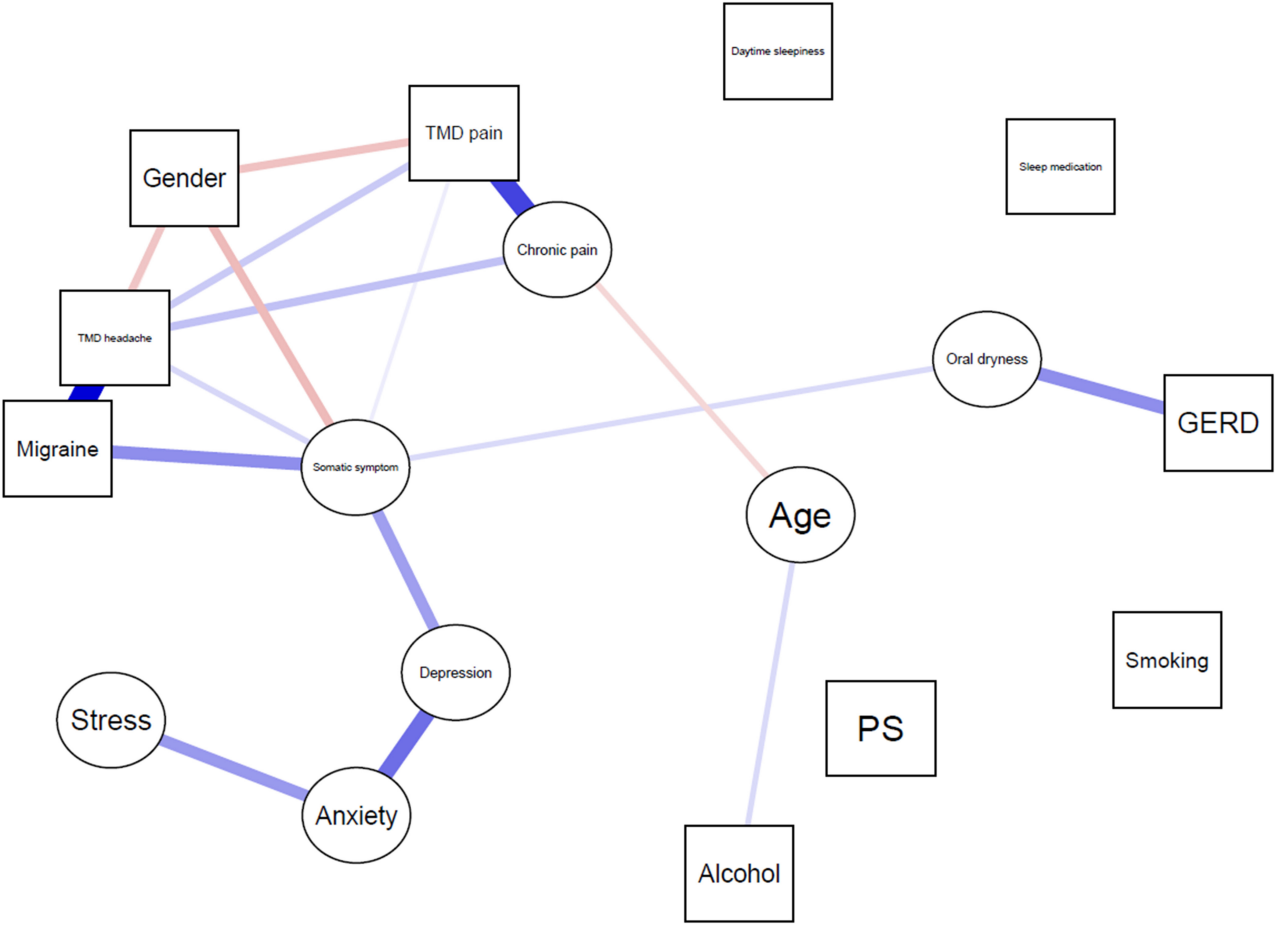
Network model of PS, demographic variables, lifestyle variables, psychological variables, pain‐related variables and sleep‐related variables. The squares represent categorical variables and the circles represent continuous variables. The blue lines represent positive associations and the red lines represent negative associations. Thicker and darker coloured lines represent stronger associations. GERD, gastroesophageal reflux disease; PS, primary snoring; TMD, temporomandibular disorder.

**FIGURE 2:**
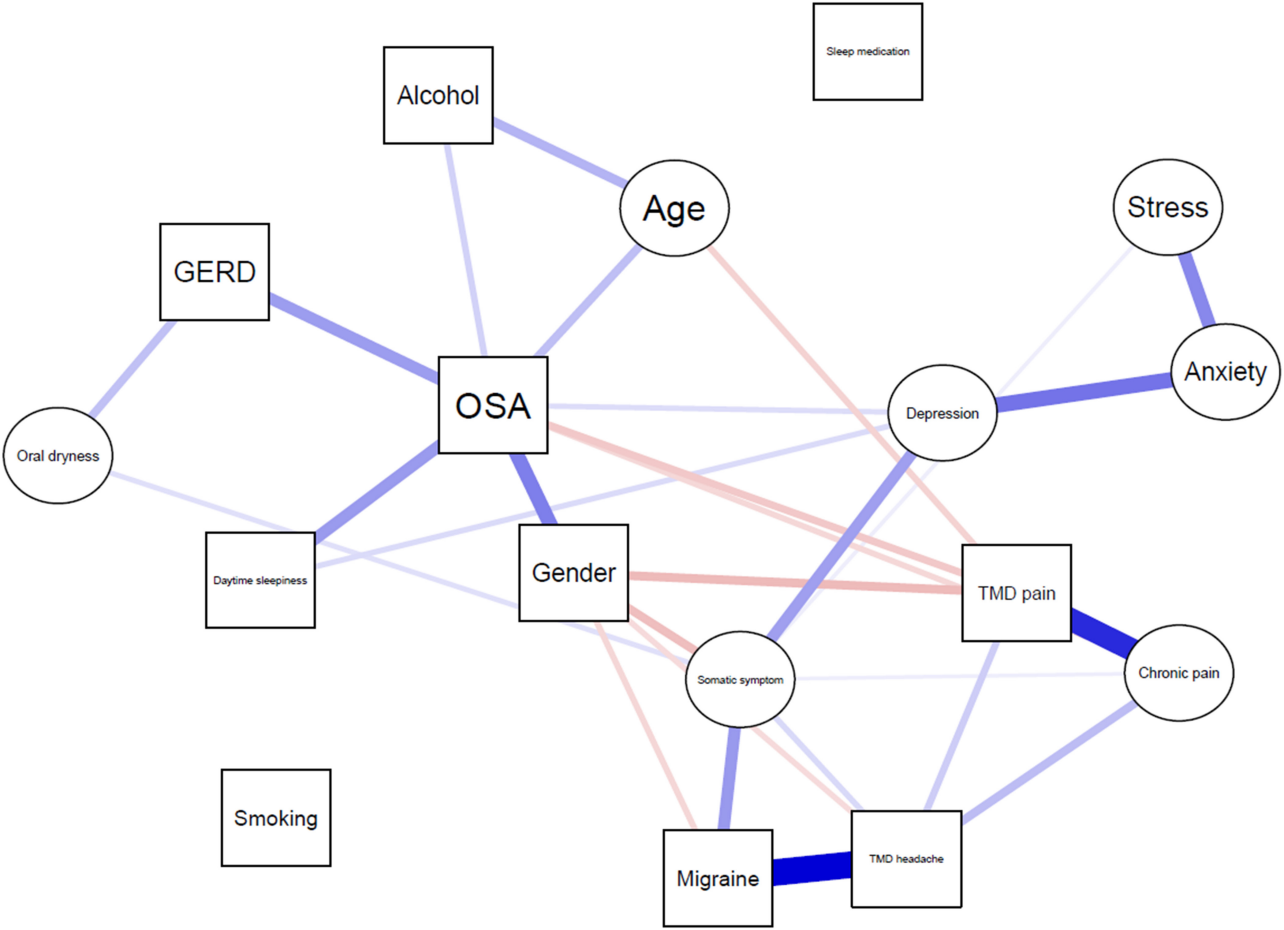
Network model of OSA, demographic variables, lifestyle variables, psychological variables, pain‐related variables and sleep‐related variables. The squares represent categorical variables and the circles represent continuous variables. The blue lines represent positive associations and the red lines represent negative associations. Thicker and darker coloured lines represent stronger associations. GERD, gastroesophageal reflux disease; OSA, obstructive sleep apnoea; TMD, temporomandibular disorder.

Results of the network analyses for PS and OSA are shown in Figures 1 and 2, respectively. For PS, network analysis confirmed that no factor was associated with PS. For OSA, it was found that age, gender, daily alcohol consumption, depression, daytime sleepiness and GERD risk were directly and positively associated with OSA, while TMD‐pain risk and chronic pain were directly and negatively associated with OSA. A list of both significant and insignificant associations in the network model for PS and OSA is shown in Appendix S1 and Appendix S2, respectively.

## 4 DISCUSSION

The present study aimed to identify associated factors of PS and OSA from self‐reported questionnaire data in SB patients. For PS, no associated factor was identified, neither in the multinomial logistic regression model nor in the network analysis. For OSA, increased age, male gender, daily alcohol consumption, depression, daytime sleepiness and high risk of GERD were found to be risk factors of OSA, while high risk of TMD pain and chronic pain were found to be protective factors of OSA, both in the multinomial logistic regression model and in the network analysis.

Notably, there was a female dominance (662 of 968; 68.4%) in the included SB patients. Regarding the prevalence of SB in men and women, previous studies reported conflicting results, i.e., some studies found that SB is more prevalent in women than in men,^28^, ^29^ while other studies reported comparable SB prevalence between men and women.^12^, ^30^ A possible explanation to the female dominance in the included SB patients is that women are more vulnerable to stress‐related disorders^31^ and self‐reported bruxism mirrors stress.^32^ In addition, due to the fact that more than half of the patients who are referred to the OPD clinic are for TMD/oro‐facial pain and females are more vulnerable to TMD than males^33^ (also confirmed in the present study; Figure 2), it is also possible that the female dominance is due to the fact that a majority of the study population is TMD/oro‐facial pain patients with a SB complaint, rather than a representative SB population. In the present study, the prevalence of PS in SB patients was 7.3%. It needs to be noted that given the high prevalence (32.7%) of snoring as a symptom in SB patients,^12^ the prevalence of PS in this study was lower than expected. A possible reason is that some primary snorers were misidentified as participants without sleep‐related breathing condition due to the ambiguous nature of the snoring question in the STOP‐Bang questionnaire, viz., ‘Do you snore loudly’? It is not unlikely that some participants did snore but nevertheless answered ‘No’ to this question as they did not consider their snoring sounds loud. Another possible reason is that it has been reported that some females underreport the fact that they snore and underestimate the loudness of their snoring sounds.^34^ Given the dominance of women in this study population, this social desirability bias^35^ may have significantly affected the results of PS‐related assessments. The above‐mentioned two potential biases may explain not only the relatively low prevalence of PS in SB patients, but also why no associated factor was identified for PS. In addition, in the present study, the prevalence of OSA in SB patients was 17.7%, which is lower than those in previous studies (20.6%–27.3%).^7^, ^10^, ^11^ This discrepancy in the OSA prevalence may also be due to the fact that the present study identified OSA patients based on self‐report, while previous studies used polysomnography (PSG), the gold standard to diagnose OSA and SB.^7^, ^36^

How increased age, male gender, alcohol and daytime sleepiness may contribute to the OSA pathophysiology has been thoroughly discussed in previous studies.^19^, ^36^ As for the other identified associated factors in the present study, depression was found to affect 35% of OSA patients,^37^ and two longitudinal studies identified OSA as an independent risk factor of depression.^38^, ^39^ Available evidence suggests that OSA may lead to the development or exacerbation of depressive symptoms by initiating or amplifying the pathologic processes of cerebral small vessel disease and blood–brain barrier dysfunction.^40^ Previous studies focusing on genetic factors also found two microRNAs (viz., miR‐15b‐5p and miR‐92b‐3p) that may modify OSA‐related depression.^41^ On the other hand, compared with individuals without SB, SB patients were found to have higher scores of depression.^42^ SB has long been known to be regulated centrally, and depression is considered a potential aetiology of SB^16^. In addition, for patients with depression, antidepressants may worsen or induce SB.^43^ Taken together, it is possible that there is a potential association between OSA and SB, in which depression functions as a connecting factor, i.e., OSA leads to the development or exacerbation of depression, and depression consequently results in SB. However, future longitudinal studies are needed to test this hypothesis.

It is noteworthy that, compared with depression, arousal is a more well‐recognised connecting factor between OSA and SB, which may better explain the association between OSA and SB at event level. A previous PSG study reported the temporal association between OSA events and SB events, and the authors hypothesized that the co‐activation of oropharyngeal muscles may maintain the patency of the airway but may lead to SB at the same time, suggesting a potential protective role of SB against airway obstruction.^42^ There is increasing evidence that SB events that are time‐linked to OSA events are associated with OSA event‐related arousals rather than with OSA events *per se*.^44^ Further, although several hypotheses on the mechanism of associations between GERD on the one hand and OSA and SB on the other hand have been proposed, arousal is more likely to play a pivotal role in the potential comorbidity network among GERD, OSA and SB, which is suggested by the present study. That is, because, in addition to SB events, reflux episodes were also found to occur in proximity with respiratory arousals and arousal was found to be a risk factor of the presence of reflux episodes.^45^ In addition, recent studies reported the effect of OSA‐related managements, such as continuous positive airway pressure (CPAP),^46^ on reducing GERD‐related symptoms. The above‐mentioned evidence suggests that arousal may be a connecting factor among GERD, OSA and SB, but future studies on patients with coexisting OSA, SB and GERD are needed to test this hypothesis.

In addition to GERD, the present study also suggested a comorbidity network among TMD pain, OSA and SB. The negative association between TMD pain and OSA in SB patients may be explained by the gender difference between participants without sleep‐related breathing condition and OSA patients: a majority (60.8%; Table 2) of OSA patients was male, while only 25.8% (Table 2) of participants without sleep‐related breathing condition was male. Given that females are more vulnerable to TMD than males,^33^ it is reasonable that the prevalence of TMD pain in participants without sleep‐related breathing condition was significantly higher than that in OSA patients, which may consequently lead to the negative association between TMD pain and OSA in the analyses. In brief, the dominance of women in participants without sleep‐related breathing condition resulted in the negative association between TMD pain and OSA. It should be noted that previous studies reported positive associations between TMD pain and OSA.^15^, ^17^, ^47^ Cunali et al.^17^ and LeResche^47^ found that patients with OSA experience more TMD pain than otherwise healthy individuals. In addition, OSA symptoms were found to precede first‐onset TMD‐pain complaints via central sensitization.^15^ In the present study, a negative association was also observed between OSA and chronic pain. This negative association may be because the participants' chronic pain scores were highly correlated to their complaints about TMD pain. This hypothesis is confirmed in the network analysis, where a strong and positive association exists between chronic pain and TMD‐pain risk.

Due to its retrospective design, this study has several limitations. Firstly, the relatively small number of events of interest (especially for PS) does not satisfy the recommended standard that events per variable (EPV) in multinomial logistic regression model should be more than 20.^48^ According to the recommended standard for multinomial logistic regression models,^48^ the category with the smallest sample size, i.e., the PS group in this study, is commonly regarded as the basis for the EPV. This suggests a minimum of 200 primary snorers, viz., 20*10 (number of variables in multivariable logistic regression model), but there were only 71 primary snorers in this study. This may have affected the results to some extent. However, the identified associated factors were explainable and the confidence intervals of associated factors were reasonable, viz., not too wide and not too narrow, suggesting that the results were reliable. Secondly, data on BMI, a frequently reported associated factor of both PS and OSA,^19^ were not collected in this study. It is not unlikely that BMI is associated with PS and/or OSA in SB population. Thirdly, as mentioned above, the majority of the study population is TMD/oro‐facial pain patients with a SB complaint, rather than a representative SB population. As a consequence, the findings may be limited to the current patient profile. Fourthly, as mentioned above, the ambiguous nature of the snoring question in the STOP‐Bang questionnaire may have led to biased results. The authors recommend future studies to diagnose SB, OSA and PS based on PSG as to confirm the findings in the present study. In addition, other factors that have been reported to be associated with PS and OSA, such as BMI and insomnia,^49^ should also be included in future studies.

## 5 CONCLUSION

Within the limitations of this study, no associated factor is identified for PS. For OSA, dentists should keep in mind that increased age, male gender, daily alcohol consumption, depression, daytime sleepiness and high GERD risk are associated with increased OSA risk in SB patients, while high TMD‐pain risk and chronic pain are associated with decreased OSA risk in this population.

## AUTHOR CONTRIBUTIONS

**Zhengfei Huang** involved in conceptualization, data acquisition, analysis and writing the original draft. **Ghizlane Aarab** involved in conceptualization and writing—review and editing. **Thiprawee Chattrattrai** involved in analysis and writing—review and editing. **Naichuan Su** involved in analysis and writing—review and editing. **Catherine MC Volgenant** involved in data acquisition and writing—review and editing. **Antonius AJ Hilgevoord and Nico de Vries involved in** writing—review & editing. **Frank Lobbezoo** involved in conceptualization, data acquisition and writing—review and editing.

## CONFLICT OF INTEREST

There is no conflict of interest.

### PEER REVIEW

The peer review history for this article is available at https://publons.com/publon/10.1111/joor.13354.

## Supporting information


Appendix S1‐S2

## Data Availability

The data that support the findings of this study are available from the corresponding author upon reasonable request.
