# Supplementary material for: Associated factors of primary snoring and obstructive sleep apnoea in patients with sleep bruxism: A questionnaire study
Source: J Oral Rehabil. 2022 Jul 12;49(10):970–9. doi: 10.1111/joor.13354 (PMC9543241; doi:10.1111/joor.13354)
Supplement: Supplementary file 1 — Appendix S1‐S2 [file JOOR-49-970-s001.docx]

**Appendices**


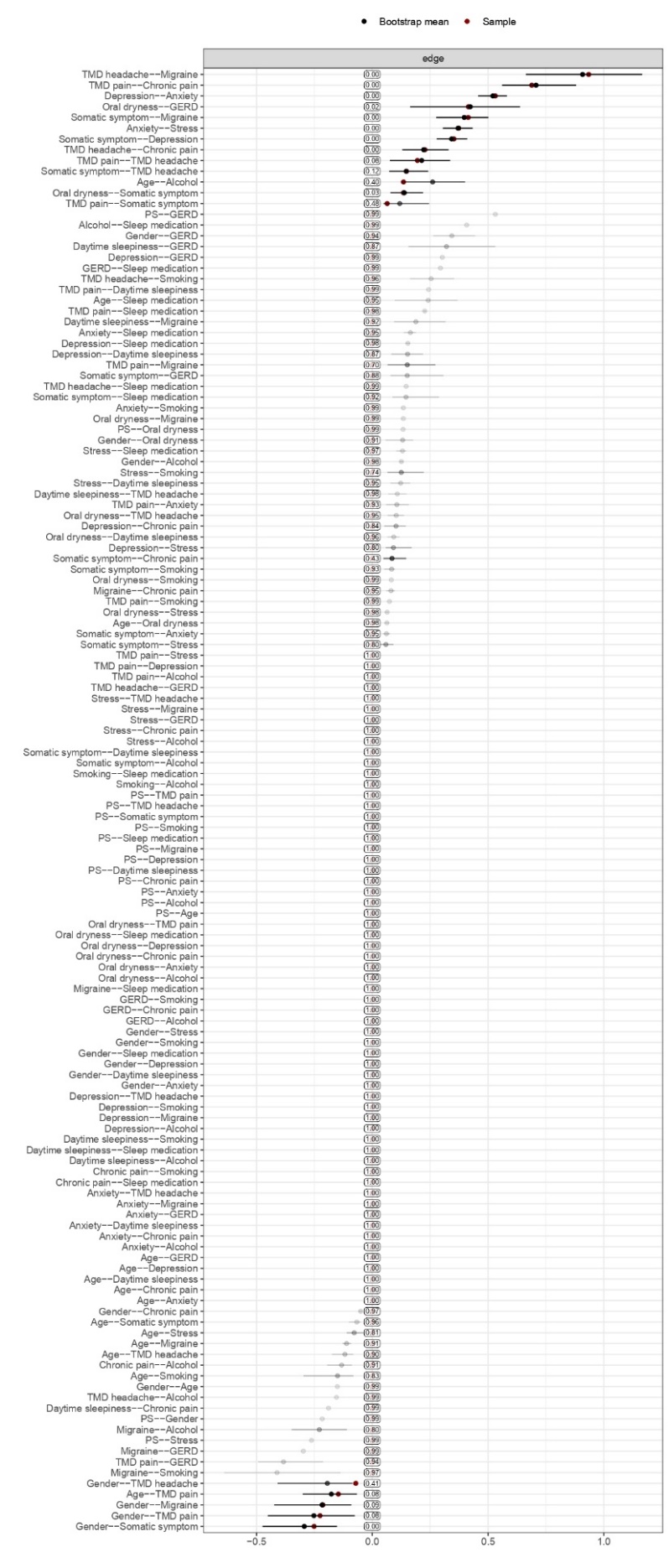


**Appendix 1.** Bootstrapped confidence intervals of the network model of PS. Only the black CIs were the edges in the network model. Grey CIs were the edges omitted from the network. The strongest edge is displayed at the top of the plot and followed by the weaker edges. The left side of the y-axis refers to negative edges, and the right side of the y-axis refers to positive edges. The overlapping bootstrapped CIs show that those edges were not significantly different from the other edges.


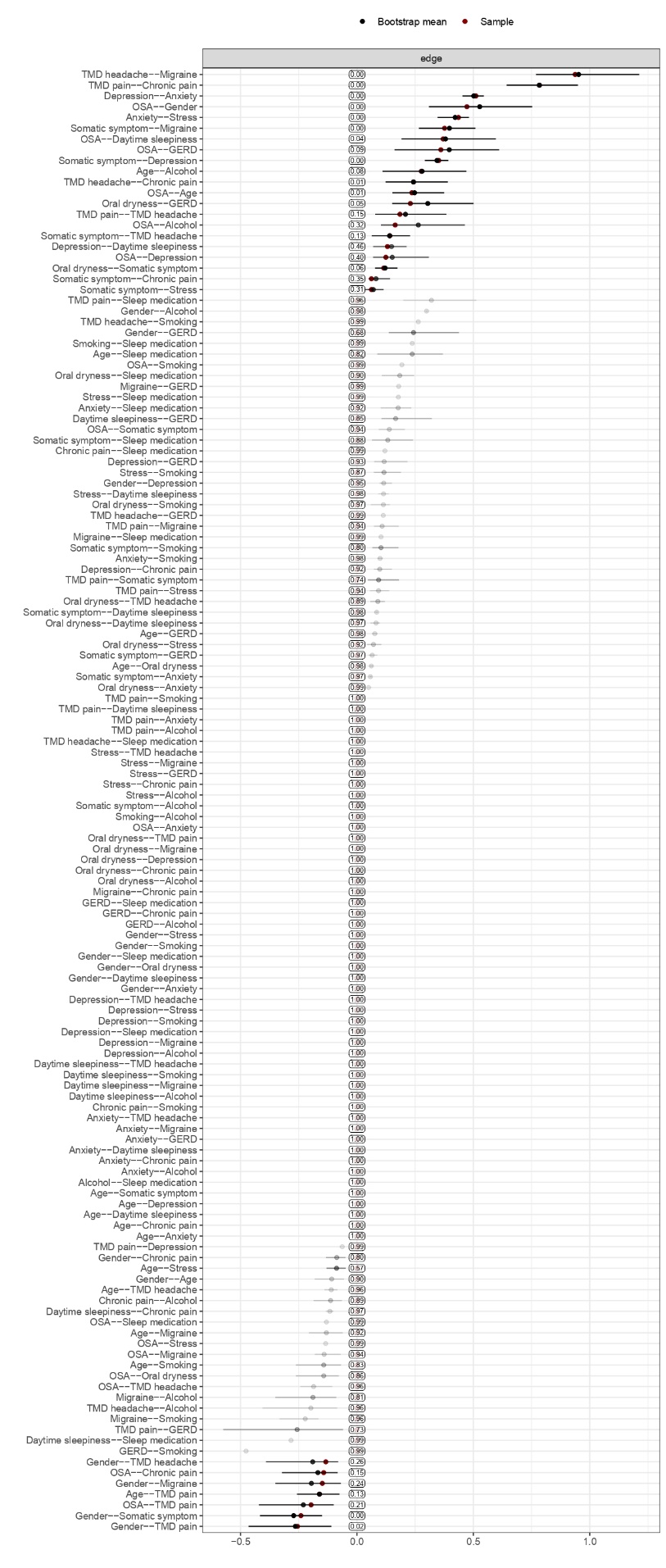


**Appendix 2.** Bootstrapped confidence intervals of the network model of OSA. Only the black CIs were the edges in the network model. Grey CIs were the edges omitted from the network. The strongest edge is displayed at the top of the plot and followed by the weaker edges. The left side of the y-axis refers to negative edges, and the right side of the y-axis refers to positive edges. The overlapping bootstrapped CIs show that those edges were not significantly different from the other edges.
